# Hamstring extensibility differences among elite adolescent and young dancers of different dance styles and non-dancers

**DOI:** 10.7717/peerj.9237

**Published:** 2020-05-26

**Authors:** Raquel Vaquero-Cristóbal, Patricia Molina-Castillo, Pedro A. López-Miñarro, Mario Albaladejo-Saura, Francisco Esparza-Ros

**Affiliations:** 1 Department of Sport Science, Catholic University San Antonio of Murcia, Murcia, Spain; 2 Department of Sport Traumatology, Catholic University San Antonio of Murcia, Murcia, Spain; 3 Department of Physical Education, University of Murcia, Murcia, Spain; 4 PhD Program, Catholic University San Antonio of Murcia, Murcia, Spain

**Keywords:** Assessment, Biomechanics, Flexibility, Health, Injury & prevention, Lower extremity, Skeletal muscle, Stretching, Training, Young

## Abstract

**Background:**

Hamstrings have been analyzed extensively due to their tendency to shorten and their effect in the lumbo-pelvic dynamics and the sagittal position of the spine in trunk flexion with extended knees positions. It has been demonstrated that practicing a certain sport results in long-term changes in hamstring extensibility. Despite this, adequate extensibility of the hamstring musculature is essential for the dancer’s performance. Several studies have found differences in the extensibility of the hamstrings depending on the dance style, but none have compared ballet, Spanish dance and modern dance. The purpose of the present research was to analyze the differences in hamstring extensibility among professional dance students based on dance style practiced and non-dancers.

**Methods:**

The sample was comprised of 210 students from the Professional Dance Conservatory (70 for ballet, 70 for Spanish dance and 70 for modern dance) and 70 non-dancers. For the assessment of hamstring extensibility, the angle in the passive and active straight leg raise (PSLR and ASLR, respectively) test, and the scores of the pelvic tilt in sit-and-reach (SR) test and the toe-touch (TT) test were randomly conducted.

**Results:**

The results showed significant differences for all the tests according to group (*p* < 0.001). In the PSLR and ASLR test, for both legs, and in the pelvic tilt in the SR test, the ballet dancers showed greater ranges of hamstring extensibility than the modern dancers and Spanish dancers (*p* ≤ 0.001). In the distance in the SR test and in the pelvic tilt in the TT test, the ballet dancers obtained higher values than the Spanish dancers (*p* = 0.004 and *p* = 0.003, respectively). The modern dancers showed higher ranges of hip flexion than the Spanish dancers in the ASLR test for both legs and in the pelvic tilt in the SR test (*p* from 0.007 to <0.001). Dancers showed significantly higher hamstring extensibility than non-dancers in all the tests (*p* < 0.001).

**Conclusions:**

The systematic practice of dance, regardless of the style, seems to lead to high ranges of hamstring extensibility. Ballet dancers have the greatest hamstring extensibility.

## Introduction

Hamstrings, comprised by three muscles, the biceps femoris, semitendinosus and semimembranosus, have been analyzed extensively due to their tendency to shorten. This tendency is caused by their histological and biomechanical characteristics (Vaquero-Cristóbal et al., 2015).

Different research studies have demonstrated that practicing a certain sport can cause reductions in hamstring extensibility (López-Miñarro, Muyor & Alacid, 2014; Muyor et al., 2014a). There is an exception in those sports where technical movements involves hamstring muscles extensibility because sport practice can induce a traction stimulus that increases hamstring extensibility. Furthermore, specific stretching programs are included in the training to achieve the range of motion which allows performing these specific movements with a good technique. This can involve a high volume of both intermittent and continuous hamstring stretching, which can increase the range of motion (Donti et al., 2018; Santonja et al., 2007).

Changes in hamstring extensibility can be mostly a consequence of changes in stretch tolerance (Avrillon et al., 2020; Marshall & Siegler, 2014; Weppler & Magnusson, 2010) and adaptations in morphological and neurological systems which reduce some reflexes and stiffness as a consequence of morphological adaptations and/or changes of the tendon material (Avrillon et al., 2020; Bohm, Mersmann & Arampatzis, 2015; Lima et al., 2019). As a consequence, hamstring stretching can induce changes in hamstring torque-angle profile, favoring a peak torque at longer muscle lengths, a large functional range of motion and a higher mechanical work (Moltubakk et al., 2016). Kokkonen et al. (2007) attributed the gain in power to increases in muscle length. However, other studies have found that an acute effect of stretching reduces power output and maximal force (Baker, Wilson & Carlyon, 1994) and no relationships between hamstring extensibility, knee strength and torque and work production has been found in dancers (Agopyan, 2018).

Great hamstring extensibility has been found in adolescents who are involved in aesthetic group gymnastics (Conesa-Ros, Martínez-Gallego & Santonja, 2016), in professional trampoline gymnasts (Sáinz de Baranda et al., 2014) and in taekwondo athletes of different ages and categories of competition (Bridge et al., 2014).

Adequate extensibility of the hamstring musculature is also essential for the dancer’s performance, because there are several dance steps in which a high hamstring extensibility is necessary for proper technical execution (Twitchett, Koutedakis & Wyon, 2009). The importance of the hamstring muscles’ extensibility in dance lies in the effect they have on the lumbo-pelvic dynamics and the sagittal position of the spine (López-Miñarro, Muyor & Alacid, 2014; McGill, 2002). A high hamstring extensibility allows reaching a maximal trunk flexion position with extended knees (López-Miñarro, Muyor & Alacid, 2014; McGill, 2002), positions that are repeated frequently during dance training, especially in ballet and modern dance (Forczek, Baena-Chicón & Vargas-Macías, 2017; Grant, 2012; Villanueva, 1995).

However, these kinds of positions can result in viscoelastic deformation of passive tissues in the posterior trunk and consequently a decrease in trunk stiffness (McGill & Brown, 1992; Shin & Mirka, 2007; Toosizadeh et al., 2012), which can cause and extra activation of trunk muscles (McCook, Vicenzino & Hodges, 2009; Shin & Mirka, 2007). It may be associated with excessive spinal loads and muscle fatigue (Shin, D’Souza & Liu, 2009), factors associated with lower back disorders (Bakker et al., 2009). In fact, the lower back and knee joints are the most frequently and strongly affected joints in dance (Lampe et al., 2019). Extreme flexibility and generalized joint laxity or hypermobility can increase the injury risk in dancer by about 22–43% (Bronner & Bauer, 2018). In spite of this, higher joint ranges of motion are required for performing most of the dance steps in different styles, so injury risk has to be assumed (Bird, 2016).

Few studies have analyzed hamstring extensibility in dancers (Agopyan, 2018; Chatfield et al., 1990; Claessens et al., 1987; Gómez-Lozano et al., 2012; Koutedakis et al., 2007; Martínez et al., 2014; Oreb et al., 2006; Steinberg et al., 2006). Furthermore, they have usually included a small sample size (with only one study including more than 35 participants in the dance group) (Steinberg et al., 2006); an absence of a control group (Agopyan, 2018; Claessens et al., 1987; Koutedakis et al., 2007; Oreb et al., 2006), or separated groups according to each dance style (Steinberg et al., 2006), although the different dance styles have different techniques which can influence hamstring extensibility (Forczek, Baena-Chicón & Vargas-Macías, 2017; Grant, 2012; Villanueva, 1995); and an absence of homogeneity in the age and the level of the dancers, although these factors can affect hamstring extensibility (Conesa-Ros, Martínez-Gallego & Santonja, 2016).

The Spanish Professional Dance Conservatory usually offers the specialization in ballet, modern dance, and Spanish dance, which includes flamenco, folklore, “escuela bolera” and Spanish stylized dance (Real Decreto, 2015). Steinberg et al. (2006) compared the hamstring extensibility in adolescent dancers from different styles in a single group. Dancers showed significantly higher hamstring extensibility than the control group. However, the validity and reliability of the test used was not been established.

As for ballet, it has been found that students from the Professional Dance Conservatory showed high hamstring extensibility (Claessens et al., 1987) and that adolescent professional dancers showed significantly higher hamstring extensibility than a recreational group (Martínez et al., 2014).

Gómez-Lozano et al. (2012) studied the hamstring extensibility of a group of professional and student flamenco and Spanish dancers in comparison with a control group. They found significant differences in favor of the dancers.

Other studies showed that modern dance students had high values (Agopyan, 2018; Koutedakis et al., 2007). Furthermore, a study compared a group of modern dancers, dividing them according to their level, with a sedentary, non-dancer group. They found that the advanced group showed significantly higher values than non-dancers and beginners (Chatfield et al., 1990).

Only one study compared dancers from different disciplines. Oreb et al. (2006) compared the hamstring extensibility of Croatian professional folklore dancers and ballet dancers, finding that the second group had greater extensibility. However, folk dance techniques differs among countries (Nielsen, 2011).

There is weak evidence about hamstring extensibility in difference dance styles during the growing stage, although it is a critical period due to the increase in muscle mass and to the structural changes that occur at this stage (Bell & Hoshizaki, 1981; Cejudo et al., 2019). Therefore, the objective of this research was to analyze the differences in hamstring extensibility among professional dance students based on dance style practiced and non-dancers.

## Materials and Methods

### Design

A retrospective, cross-sectional and observational study was conducted.

The study design and manuscript development were focused following the Strengthening the reporting of observational studies in epidemiology (STROBE) statement (Vandenbroucke et al., 2014).

Before beginning the study, an authorization was obtained from the institutional bioethics committee (Catholic University of Murcia ethical committee; number: 20-09-2013).

The calculations for establishing the sample size were performed using Rstudio 3.15.0 software. The significance level was set at α = 0.05. The standard deviation (SD) was established based on previous studies for the passive straight leg raise (PSLR) test (mean SD = 16.67) (Gómez-Lozano et al., 2012; Koutedakis et al., 2007); active straight leg raise (ASLR) test (mean SD = 18.7) (Agopyan, 2018); score in sit-and-reach (SR) test (mean SD = 4.24) (Claessens et al., 1987; Gómez-Lozano et al., 2012; Oreb et al., 2006); pelvic tilt in SR test (mean SD = 17.6) (Gómez-Lozano et al., 2012); score in toe-touch (TT) test (mean SD = 7.28) (González-Gálvez, Marcos-Pardo & Carrasco-Poyatos, 2019); and pelvic tilt in TT test (mean SD = 12.68) (Chatfield et al., 1990). With an estimated error (*d*) of 3.91° in PLSR, 4.38° in ALSR, 0.99 cm in the score of the SR test, 4.11° in the pelvic tilt of the SR test, 1.70 cm in the score of the TT test and 2.96° in the pelvic tilt of the TT test, a valid sample size for a confidence interval (CI) of 95% was 70 for each group.

### Participants

**Figure 1:**
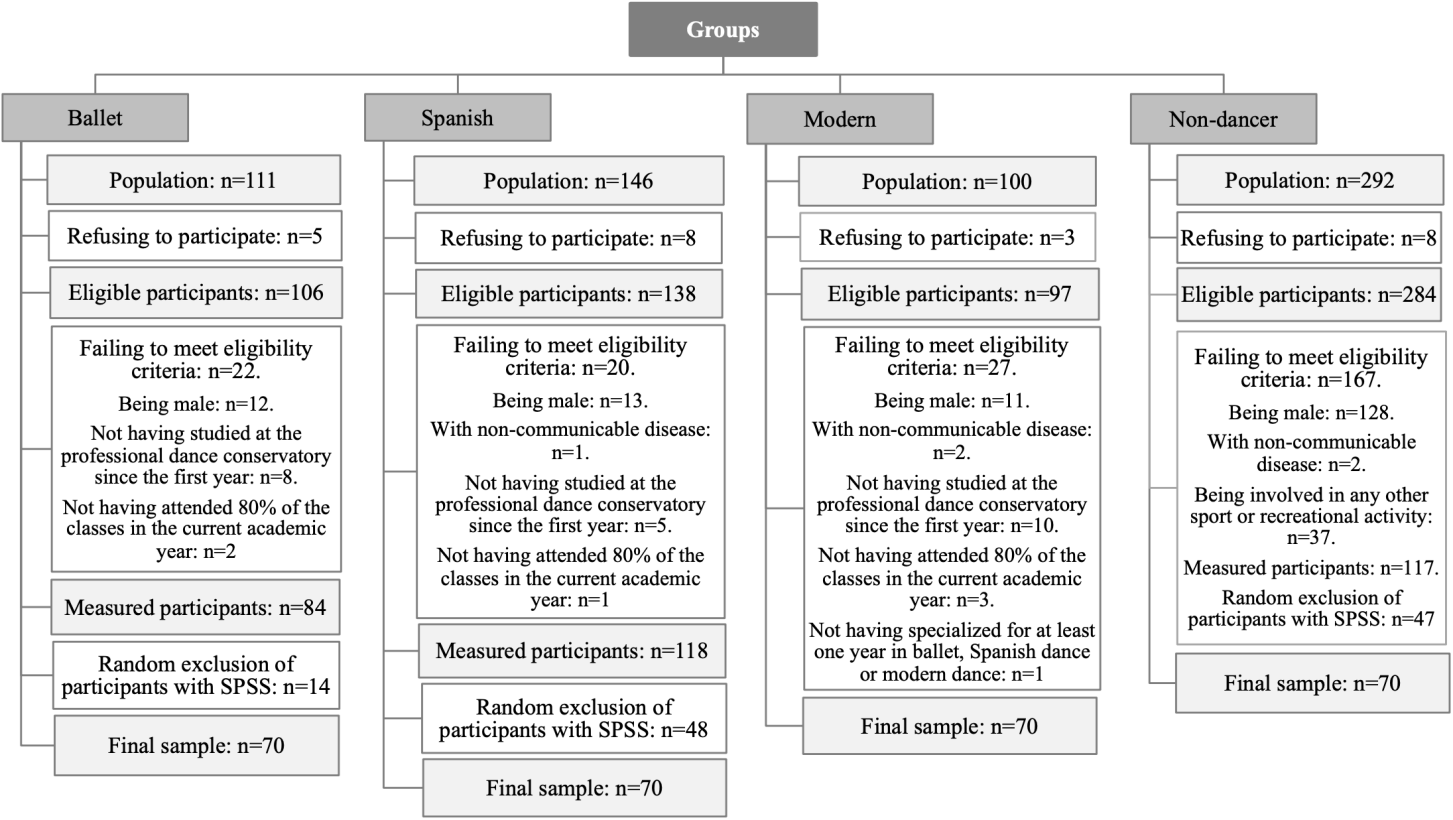
Consort flow diagram.

The participant consort flow diagram is shown in Fig. 1. The present study was conducted with a sample of 210 female students from the Professional Dance Conservatory and 70 non-dancer girls. The Professional Dance Conservatory is an official Professional Dance Conservatory school where dancers train to be professional dancers for ten years. They receive a base of all the dance styles during the first four years and they specialize in a style from the 5th to 10th years. Classes always comprise less that ten practitioners, with usually one group per dance style each academic year. Dancers of the present study were all students in their 6th to 10th academic year in the Conservatory. They train between three and 5 h per day, 5 days per week. The dancers were divided into three natural groups according to the dance style practiced (70 studied ballet, 70 Spanish dance and 70 modern dance). The inclusion criteria were: (a) being female; and (b) not having a non-communicable disease. Specific inclusion criteria in the dance group were: (c) having studied at the Professional Dance Conservatory since the 1st year consecutively; (d) having attended 80% of the classes in the current academic year; and (e) having specialized for at least one year in ballet, Spanish dance or modern dance. The specific inclusion criteria in the non-dancer group was: (c) not being involved in sports or a recreational activity for more than 30 min, three times a week.

### Procedure

The participants were informed of the procedures and an informed consent was given to them. The consent was signed by the participants if they of legal age or by their parents or tutors if they were not. Data were collected between 2013 and 2017.

For the assessment of hamstring extensibility, a PSLR, an ASLR, a SR and a TT test were conducted in random order. Randomization was performed with Randomizer software (Medical University of Graz, Austria). The participants did not perform any warm-up exercises before the tests to not influence the variables measured (Díaz-Soler et al., 2015; Ortega, Menéndez & Herrero, 2017). The participants should not have had intense exercise the day before, nor had exercise or stretching sessions on the day of the assessment. The temperature of the room was standardized to 24 °C and all the measurements were performed from 9:00 to 14:00. All the tests were carried out by the same researcher and one assistant. Both were blinded about the dance style practiced by the dancers and the academic year that dancers and non-dancers belong to.

### Hamstring assessment

**Figure 2:**
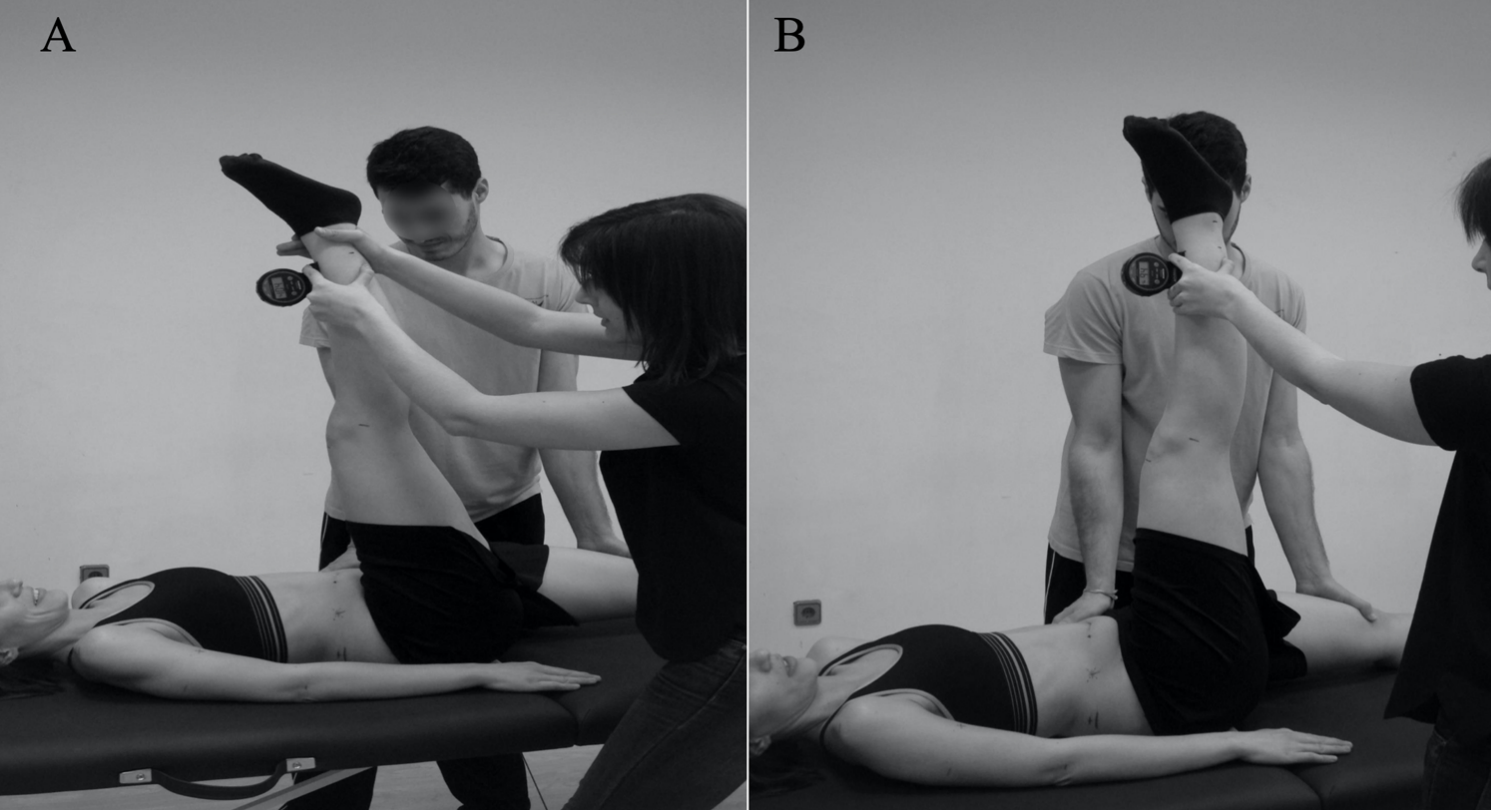
Hip flexion angle measured during passive (A) and active (B) straight leg raise test.

To perform the ASLR test, the participants laid in the supine position on a table and maintained the unexamined leg in contact with the table. A trapezoid shaped device formed by a layer of rigid plastic and a padded layer in contact with the participant, with a small protrusion in the area to be placed in the lumbar curvature, which was placed below the lumbar area to prevent flattening of the area while the test was being conducted (Lumbosant, Ortopedia Murcia, Spain). They were asked to perform the test with the knee extended and the ankle in the maximal dorsal flexion. The subjects were asked to actively flex the coxofemoral joint of the examined leg. To perform the PSLR test, the protocol followed was similar to the ASLR test, although it was the researcher who elevated the measured leg of the participants to reach the maximum range of motion of the coxofemoral joint (Fig. 2). The measurements were registered with a Uni-level digital inclinometer (Isomed, CA, USA). The end point for both straight leg raise (SLR) tests was determined by 1 or both criteria: (a) the participants reported pain in the hamstring muscle and/or (b) palpable onset of pelvic rotation.

The references provided by Sáinz de Baranda et al. (2005) and López-Miñarro et al. (2008) were followed to categorize the participants according to their values from the PSLR test. Values considered normal in the PSLR test were ≥75°, with shortness grade I (74°–61°) and shortness grade II (60° or less). SLR has been used as the gold standard for the analysis of hamstring extensibility (Muyor et al., 2014a, 2014b; Sáinz de Baranda et al., 2014). The test has shown a good reliability with an interclass correlation coefficient (ICC) from 0.87 to 0.95 (Ayala et al., 2012b; Cejudo et al., 2015). Muyor et al. (2014b) found an ICC ranging from 0.85 to 0.98 between PSLR and ASLR in adolescent boys and girls, which suggests that both can be used indistinctly.

**Figure 3:**
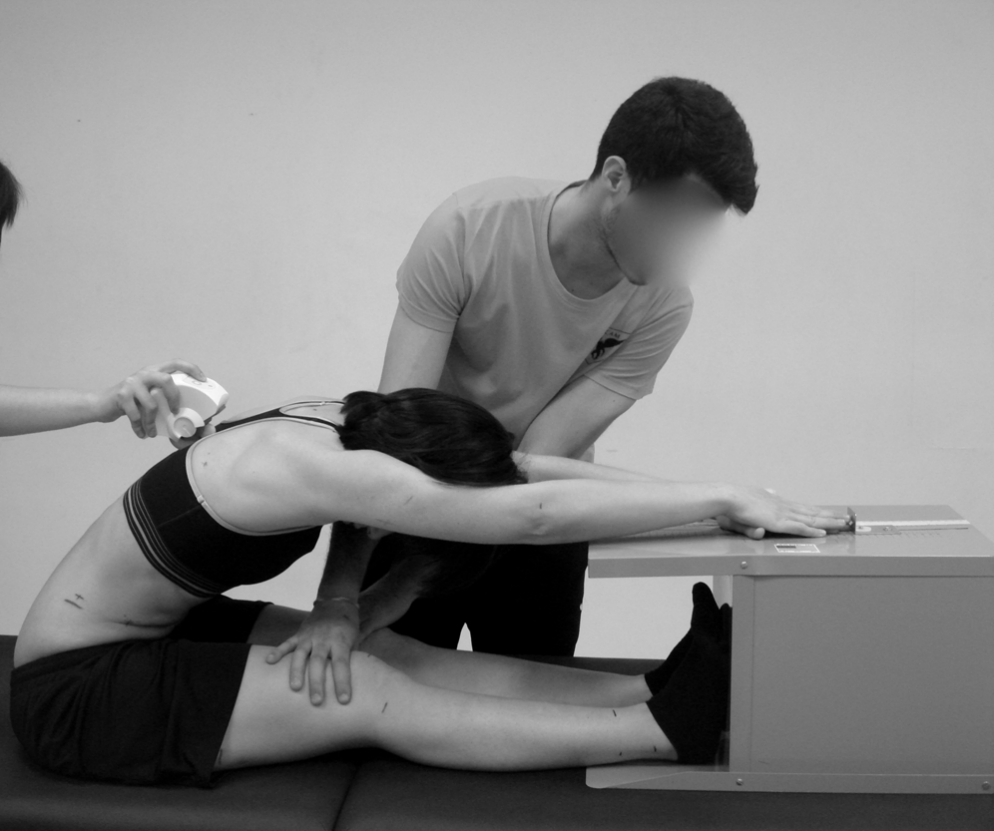
Distance and pelvic tilt measured during maximal trunk flexion in sitting with the extended knees (sit-and-reach test).

**Figure 4:**
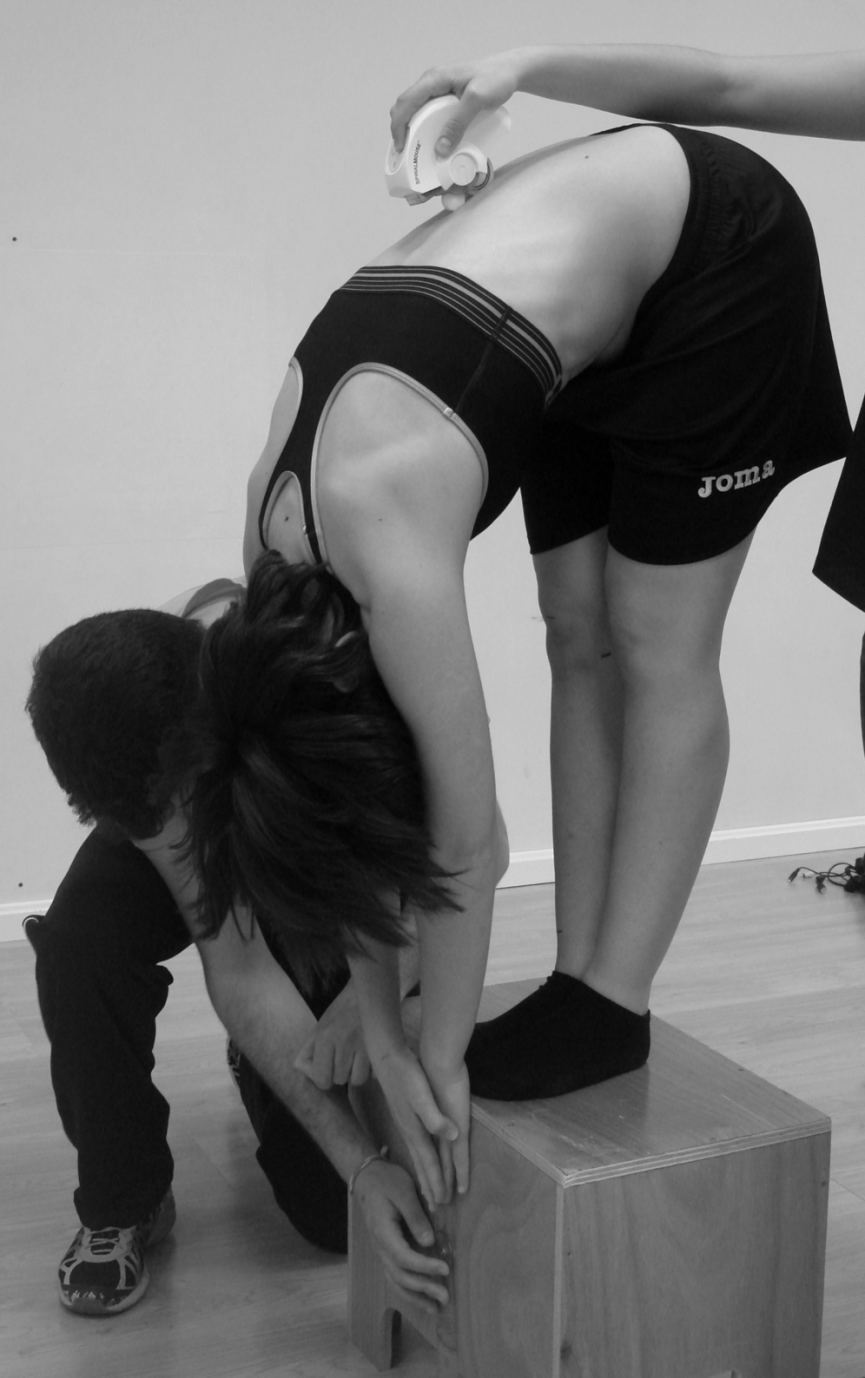
Distance and pelvic tilt measured during maximal trunk flexion in standing with the extended knees (toe-touch test).

For the assessment of the SR test, the participants were asked to sit with the soles of the feet in contact with an Acuflex Tester III test box (Novel Products, USA) with the knees extended (Fig. 3). The TT test was conducted with the participants standing on both feet on the Acuflex Tester III test box (Novel Products, IL, USA) and flexing the torso with the knees extended (Fig. 4). In both tests the participants were asked to reach the maximum distance possible with the distal phalanges. A value of zero meant that the individual reached the level of the feet, whereas a negative value meant that this level was not reached and a positive value meant that this level was surpassed. The participants were considered to have a normal hamstring extensibility in the SR test when reaching a distance greater than −2 cm, shortness grade I between −3 and −9 cm and shortness grade II with values less than or equal to −10 cm (López-Miñarro et al., 2008). In the distance of the TT test, normal values were established as equal to or greater than −4 cm, shortness grade I between −5 and −11 cm and shortness grade II for values equal to or less than −12 cm (Rodríguez-García & Santonja, 2001). Some meta-analyses has shown that the scores in the SR and TT tests have a moderate validity to measure the hamstring extensibility (Mayorga-Vega, Merino-Marban & Viciana, 2014; Mayorga-Vega et al., 2013). As for the reliability, both scores in the SR and TT tests have shown to have a good ICC (0.98 and 0.89, respectively) (Ayala et al., 2012a; López-Miñarro, Andújar & Rodríguez-García, 2009).

Besides measuring the distance reached with the box, the SR and TT test evaluated the pelvic tilt with the Spinal Mouse^®^ (Idiag, Switzerland) (Figs. 3 and 4, respectively). The Spinal Mouse^®^ was chosen as it is a non-invasive, reproducible, valid and reliable technique for measuring the sagittal profile of the thoracic and lumbar spinal column and pelvic tilt (Carlucci, Chiu & Cilifford, 2001), with a reliability (ICC 95%) from 0.85 to 0.98 in the sagittal plane (Topalidou et al., 2014) and a validity in pelvic tilt with respect to radiographic measurement of 0.86 (Guermazi et al., 2006).

In the SR test, a value in the pelvic tilt of 0° corresponded to a vertical position, so positive values corresponded to anterior pelvic tilt and negative values corresponded to posterior pelvic tilt. In the TT test, a value of 90° indicated that the pelvis was in neutral position, with greater angles indicating the presence of anterior pelvic tilt and lower values indicating the presence of posterior pelvic tilt. In the tilt of the SR test, values were considered normal up to −19°, shortness grade I between −20° and −27°, and shortness grade II when the angle was greater than or equal to −28° (López-Miñarro et al., 2008). In the tilt of the TT test, values greater than or equal to 65° were considered normal, and considered shortness when the value was equal to or less than 64° (Rodríguez-García & Santonja, 2001). The pelvic tilt in the SR and TT tests has demonstrated to have a good validity to measure hamstring extensibility with an ICC near to 0.80 (Sáinz de Baranda et al., 2014). About the reliability, both pelvic tilt in SR and TT has shown a good ICC (0.93, respectively) (López-Miñarro, Andújar & Rodríguez-García, 2009).

### Statistical analysis

The data distribution was initially evaluated with the Kolmogorov–Smirnov normality test. All the hamstring extensibility variables had a homogeneous distribution but not the demographic variables. Levene’s test was used to check the homogeneity of variances and the Mauchly sphericity test was used to check the sphericity assumption. The mean values and standard deviation of the different variables were obtained with descriptive statistics.

A Kruskal–Wallis *H* test was conducted to analyze the differences between groups in demographic variables. If significant differences between groups were found, a pairwise comparison was performed using the Mann–Whitney *U* test. The level of statistical significance was established *p* < 0.05. A one-way analysis of covariance (ANCOVA), with demographic variables as covariables (age, weight, height and body mass index (BMI)), was performed to determine the differences in the hamstring extensibility test between the dancers of the different dance styles and the non-dancer group. The significance of the repeated multivariate measurements was confirmed by Wilk’s lambda, Pillai’s trace, the Hotelling trace and Roy’s tests, all of which obtained similar results. The level of statistical significance was established beforehand at α < 0.05. If significant differences between groups were found, a pairwise comparison was performed using the Bonferroni correction for multiple comparisons (*p* < 0.0125). The effect size was calculated using Cohen’s d coefficient. A value lower than 0.2 was considered low effect size; a value between 0.2 and 0.4 was considered a low-moderate effect; a value between 0.4 and 0.6 was considered a moderate effect; a value between 0.6 and 0.8 was considered moderate-high effect; and a value higher than 0.8 was considered a high effect (Cohen, 1988). A χ^2^ test was performed to analyze the difference among participants in the classification of hamstring extensibility. The analysis was conducted with the Statistical Package for the Social Sciences (SPSS) (version 21.0, IBM, USA).

## Results

The characteristics of the sample are presented in Table 1. The mean and standard deviation of the analyzed variables is shown in the Table 2 and the inter-group differences in Table 3. Significant differences were found for all the tests according to the group. The subsequent Bonferroni adjustment revealed that the differences were between the ballet dancers and both the Spanish dancers and the modern dancers, with higher ranges for the former in the PSLR and ASLR tests for both legs (*p* < 0.001) and in the pelvic tilt of the SR test (*p* < 0.001 with respect to Spanish dance; *p* = 0.001 with respect to modern dance), with a moderate to high size effect. In regard to the distance of the SR test and pelvic tilt of the TT test, the ballet dancers were found to have higher values than those of the Spanish dancers (*p* = 0.004 and *p* = 0.003, respectively), with a high size effect, without significant differences with those practicing modern dance (*p* from 0.045 to 1.000). Non-significant differences were found between ballet and the other dance styles with regard to the distance of the TT test after Bonferroni correction for multiple comparisons was applied (*p* from 0.017 to 0.045).

**Table 1:** Characteristics of the participants.

	Ballet (*n* = 70)	Spanish (*n* = 70)	Contemporary (*n* = 70)	Non-dancer group (*n* = 70)	*p* Values
Age (years)	16.98 ± 1.73	17.20 ± 2.32	17.42 ± 2.18	17.41 ± 1.02	*p* = 0.174
Weight (kg)	52.48 ± 6.12	54.84 ± 6.98	54.82 ± 7.46	54.03 ± 9.80	*p* = 0.166
Height (cm)	161.13 ± 6.49	161.20 ± 5.79	161.13 ± 7.40	159.92 ± 4.96	*p* = 0.492
BMI (kg/m^2^)	20.17 ± 1.50	21.08 ± 2.30	21.03 ± 1.84*	21.18 ± 4.13	*p* = 0.020
Academic year (years)	8.07 ± 1.11	8.14 ± 1.36	8.37 ± 1.39	–	*p* = 0.360
Training per week (hours)	18.89 ± 2.07	18.26 ± 2.57	18.93 ± 2.90	–	*p* = 0.057
Sessions per week (days)	5.00 ± 0.00	5.00 ± 0.00	5.00 ± 0.00	–	*p* = 1.000

**Notes:**

* *p* = 0.024 in regard to ballet.

BMI, body mass index.

**Table 2:** Mean ± standard deviation of hamstring extensibility depending on the dance style practiced or non-practiced.

	Ballet (*n* = 70)	Spanish (*n* = 70)	Modern (*n* = 70)	Non-dancer group (*n* = 70)	*F* and *p* values
Right PSLR (°)	155.18 ± 17.71	130.01 ± 10.74	136.09 ± 19.78	87.55 ± 9.11	*F* = 110.07; *p* < 0.001
Left PSLR (°)	152.17 ± 16.84	126.10 ± 11.88	132.81 ± 18.24	89.20 ± 9.69	*F* = 97.98; *p* < 0.001
Right ASLR (°)	123.11 ± 11.61	101.71 ± 9.18	110.66 ± 16.11	72.51 ± 10.91	*F* = 95.69; *p* < 0.001
Left ASLR (°)	118.54 ± 10.73	96.42 ± 8.74	106.83 ± 13.91	73.37 ± 9.49	*F* = 94.63; *p* < 0.001
Pelvic tilt SR (°)	29.35 ± 7.15	20.14 ± 7.45	24.69 ± 10.19	−8.35 ± 8.85	*F* = 137.48; *p* < 0.001
Distance SR (cm)	19.02 ± 4.37	15.27 ± 3.54	17.95 ± 5.14	−6.42 ± 7.32	*F* = 154.82; *p* < 0.001
Pelvic tilt TT (°)	112.65 ± 6.82	104.92 ± 6.75	109.01 ± 9.93	68.78 ± 10.74	*F* = 163.72; *p* < 0.001
Distance TT (cm)	19.37 ± 4.26	15.31 ± 3.28	17.91 ± 4.77	−7.47 ± 7.73	*F* = 170.59; *p* < 0.001

**Note:**

PSLR, passive straight leg raise test; ASLR, active straight leg raise test; SR, sit-and-reach test; TT, toe-touch test.

**Table 3:** Differences inter-groups.

	Test	Mean difference	95% CI	*p* Value	Effect size
Ballet-Non-dancer	Right PSLR	68.25 ± 2.58	[61.37–75.12]	*p* < 0.001	*d* = 4.80
	Left PSLR	63.15 ± 2.52	[56.44–69.87]	*p* < 0.001	*d* = 4.58
	Right ASLR	51.17 ± 2.10	[45.60–56.74]	*p* < 0.001	*d* = 4.49
	Left ASLR	45.27 ± 1.87	[40.28–50.27]	*p* < 0.001	*d* = 4.46
	Pelvic tilt SR	39.18 ± 1.39	[35.47–42.89]	*p* < 0.001	*d* = 4.68
	Distance SR	25.37 ± 0.91	[22.93–27.81]	*p* < 0.001	*d* = 4.22
	Pelvic tilt TT	44.53 ± 1.50	[40.53–48.53]	*p* < 0.001	*d* = 4.87
	Distance TT	27.04 ± 0.91	[24.61–29.47]	*p* < 0.001	*d* = 4.30
Spanish-Non-dancer	Right PSLR	42.95 ± 2.56	[36.14–49.76]	*p* < 0.001	*d* = 4.26
	Left PSLR	37.14 ± 2.50	[30.48–43.79]	*p* < 0.001	*d* = 3.40
	Right ASLR	29.76 ± 2.08	[24.24–35.29]	*p* < 0.001	*d* = 2.89
	Left ASLR	23.15 ± 1.86	[18.20–28.20]	*p* < 0.001	*d* = 2.53
	Pelvic tilt SR	29.22 ± 1.38	[25.55–32.90]	*p* < 0.001	*d* = 3.48
	Distance SR	21.66 ± 0.91	[19.25–24.08]	*p* < 0.001	*d* = 3.77
	Pelvic tilt TT	36.45 ± 1.49	[32.49–40.42]	*p* < 0.001	*d* = 4.03
	Distance TT	22.93 ± 0.91	[20.52–25.35]	*p* < 0.001	*d* = 3.84
Modern-Non-dancer	Right PSLR	48.76 ± 2.60	[41.83–55.68]	*p* < 0.001	*d* = 3.15
	Left PSLR	43.62 ± 2.54	[36.85–50.39]	*p* < 0.001	*d* = 2.99
	Right ASLR	38.60 ± 2.11	[32.98–44.21]	*p* < 0.001	*d* = 2.77
	Left ASLR	33.25 ± 1.89	[28.22–38.28]	*p* < 0.001	*d* = 2.81
	Pelvic tilt SR	33.71 ± 1.40	[29.97–37.45]	*p* < 0.001	*d* = 3.46
	Distance SR	24.32 ± 0.92	[21.87–26.78]	*p* < 0.001	*d* = 3.87
	Pelvic tilt TT	40.51 ± 1.51	[36.48–44.54]	*p* < 0.001	*d* = 3.89
	Distance TT	25.52 ± 0.92	[23.07–27.97]	*p* < 0.001	*d* = 3.95
Ballet-Spanish	Right PSLR	25.29 ± 2.55	[18.52–32.07]	*p* < 0.001	*d* = 1.72
	Left PSLR	26.02 ± 2.49	[19.40–32.63]	*p* < 0.001	*d* = 1.79
	Right ASLR	21.41 ± 2.06	[15.91–26.90]	*p* < 0.001	*d* = 2.04
	Left ASLR	22.12 ± 1.85	[17.20–27.04]	*p* < 0.001	*d* = 2.26
	Pelvic tilt SR	9.95 ± 1.37	[6.30–13.61]	*p* < 0.001	*d* = 1.26
	Distance SR	3.71 ± 0.90	[1.31–6.11]	*p* = 0.004	*d* = 0.94
	Pelvic tilt TT	8.07 ± 1.48	[4.13–12.02]	*p* = 0.017	*d* = 1.14
	Distance TT	4.10 ± 0.90	[1.71–6.50]	*p* = 0.003	*d* = 1.07
Ballet-Modern	Right PSLR	19.49 ± 2.56	[12.67–26.30]	*p* < 0.001	*d* = 1.02
	Left PSLR	19.53 ± 2.50	[12.87–26.19]	*p* < 0.001	*d* = 1.10
	Right ASLR	12.57 ± 2.08	[7.04–18.10]	*p* < 0.001	*d* = 0.89
	Left ASLR	12.02 ± 1.86	[7.07–16.97]	*p* < 0.001	*d* = 0.94
	Pelvic tilt SR	5.46 ± 1.38	[1.79–9.14]	*p* = 0.001	*d* = 0.53
	Distance SR	1.05 ± 0.91	[−1.36 to 3.46]	*p* = 1.000	*d* = 0.21
	Pelvic tilt TT	4.02 ± 1.49	[0.05–7.99]	*p* = 0.045	*d* = 0.43
	Distance TT	1.52 ± 0.91	[−0.89 to 3.93]	*p* = 0.567	*d* = 0.32
Spanish- Modern	Right PSLR	−5.81 ± 2.54	[−12.56 to 0.95]	*p* = 0.139	*d* = −0.37
	Left PSLR	−6.48 ± 2.48	[−13.09 to 0.11]	*p* = 0.057	*d* = −0.44
	Right ASLR	−8.83 ± 2.06	[−14.31 to −3.35]	*p* < 0.001	*d* = −0.68
	Left ASLR	−10.10 ± 1.84	[−15.01 to −5.19]	*p* < 0.001	*d* = −0.90
	Pelvic tilt SR	−4.48 ± 1.37	[−8.13 to −0.84]	*p* = 0.007	*d* = −0.51
	Distance SR	−2.66 ± 0.90	[−5.05 to −0.26]	*p* = 0.021	*d* = −0.62
	Pelvic tilt TT	−4.05 ± 1.48	[−7.99 to 0.12]	*p* = 0.039	*d* = −0.48
	Distance TT	−2.58 ± 0.90	[−4.97 to −0.19]	*p* = 0.027	*d* = −0.64

**Note:**

PSLR, passive straight leg raise test; ASLR, active straight leg raise test; SR, sit-and-reach test; TT, toe-touch test.

Moreover, the modern dancers showed higher values in the ASLR test for both legs and in pelvic tilt of the SR test, with respect to Spanish dancers (*p* from 0.007 to <0.001), with a moderate to moderate-high size effect.

Dancers specialized in ballet, Spanish dance or modern dance showed higher hamstring extensibility that the non-dancer group in all the tests performed (*p* < 0.001), with a high effect.

Age showed to be a significant covariable for pelvic tilt in SR (*F* = 14.075; *p* < 0.001); while age, weight, height or BMI were not significant as covariables for the rest of measurements.

The correlation between demographic and extensibility variables are shown in Table 4. A positive correlation was found between age and BMI (*p* = 0.007) but no significant correlations were observed between the demographic data and extensibility tests. According to groups, Ballet dancers showed a negative correlation between BMI and PSLR for the left leg and ASLR for both legs (*p* from 0.011 to 0.019). Spanish dancers had positive correlations between age and BMI (*p* = 0.013) and between both age and BMI and the pelvic tilt in SR (*p* from 0.006 to 0.047). Modern dancers showed positive correlations between age and ASLR for the left leg and the pelvic tilt in SR (*p* from 0.009 to 0.015). Non-dancers presented a positive correlation between BMI and the pelvic tilt in SR (*p* = 0.001).

**Table 4:** Correlation between demographic and extensibility variables.

		BMI	Right PSLR	Left PSLR	Right ASLR	Left ASLR	Pelvic tilt SR	Distance SR	Pelvic tilt TT	Distance TT
Total (*n* = 280)	Age	*r* = 0.160 *p* = 0.007	*r* = −0.004 *p* = 0.941	*r* = −0.027 *p* = 0.653	*r* = −0.021 *p* = 0.730	*r* = −0.018 *p* = 0.765	*r* = 0.063 *p* = 0.293	*r* = −0.049 *p* = 0.414	*r* = −0.018 *p* = 0.760	*r* = −0.024 *p* = 0.695
	BMI		*r* = −0.092 *p* = 0.124	*r* = −0.109 *p* = 0.068	*r* = −0.095 *p* = 0.113	*r* = −0.102 *p* = 0.088	*r* = 0.029 *p* = 0.630	*r* = −0.082 *p* = 0.169	*r* = −0.028 *p* = 0.640	*r* = −0.064 *p* = 0.286
Ballet (*n* = 70)	Age	*r* = 0.152 *p* = 0.210	*r* = 0.120 *p* = 0.321	*r* = 0.065 *p* = 0.590	*r* = −0.024 *p* = 0.844	*r* = −0.024 *p* = 0.851	*r* = 0.148 *p* = 0.221	*r* = 0.068 *p* = 0.578	*r* = 0.205 *p* = 0.089	*r* = 0.160 *p* = 0.186
	BMI		*r* = −0.212 *p* = 0.079	*r* = −0.279 *p* = 0.019	*r* = −0.301 *p* = 0.011	*r* = −0.288 *p* = 0.016	*r* = −0.157 *p* = 0.194	*r* = −0.029 *p* = 0.815	*r* = −0.072 *p* = 0.553	*r* = −0.002 *p* = 0.988
Spanish (*n* = 70)	Age	*r* = 0.296 *p* = 0.013	*r* = −0.113 *p* = 0.351	*r* = −0.140 *p* = 0.247	*r* = −0.094 *p* = 0.437	*r* = −0.129 *p* = 0.286	*r* = 0.325 *p* = 0.006	*r* = −0.111 *p* = 0.360	*r* = −0.019 *p* = 0.876	*r* = 0.038 *p* = 0.756
	BMI		*r* = 0.148 *p* = 0.220	*r* = 0.080 *p* = 0.512	*r* = −0.126 *p* = 0.301	*r* = −0.165 *p* = 0.172	*r* = 0.238 *p* = 0.047	*r* = −0.022 *p* = 0.858	*r* = 0.162 *p* = 0.182	*r* = −0.001 *p* = 0.997
Modern (*n* = 70)	Age	*r* = 0.218 *p* = 0.070	*r* = 0.215 *p* = 0.074	*r* = 0.228 *p* = 0.058	*r* = 0.193 *p* = 0.109	*r* = 0.290 *p* = 0.015	*r* = 0.311 *p* = 0.009	*r* = 0.104 *p* = 0.391	*r* = 0.200 *p* = 0.097	*r* = 0.200 *p* = 0.097
	BMI		*r* = 0.038 *p* = 0.757	*r* = 0.087 *p* = 0.475	*r* = 0.092 *p* = 0.448	*r* = 0.175 *p* = 0.147	*r* = 0.155 *p* = 0.199	*r* = −0.027 *p* = 0.823	*r* = 0.091 *p* = 0.453	*r* = 0.107 *p* = 0.379
Non-dancer group (*n* = 70)	Age	*r* = 0.028 *p* = 0.819	*r* = 0.225 *p* = 0.061	*r* = −0.022 *p* = 0.856	*r* = 0.147 *p* = 0.225	*r* = −0.009 *p* = 0.939	*r* = 0.043 *p* = 0.723	*r* = −0.119 *p* = 0.325	*r* = −0.151 *p* = 0.212	*r* = −0.153 *p* = 0.205
	BMI		*r* = 0.052 *p* = 0.669	*r* = −0.025 *p* = 0.838	*r* = 0.114 *p* = 0.349	*r* = 0.069 *p* = 0.571	*r* = 0.399 *p* = 0.001	*r* = −0.027 *p* = 0.827	*r* = 0.150 *p* = 0.215	*r* = 0.007 *p* = 0.954

**Note:**

PSLR, passive straight leg raise test; ASLR, active straight leg raise test; SR: sit-and-reach test; TT, toe-touch test.

The number of cases and frequencies of each hamstring extensibility category in the different tests and the result of the contingency coefficient are presented in Table 5. By classifying the dancers according to normal values, it was found that all the dancers had normal extensibility in all the tests. When the group of non-dancers were classified, a moderate percentage of shortness was found. Significant differences were found in all the tests.

**Table 5:** Percentage and number of participants in each category of hamstring extensibility.

		Ballet (*n* = 70)	Spanish (*n* = 70)	Contemporary (*n* = 70)	Non-dancer group (*n* = 70)	χ^2^ and *p* values
Right PSLR	Normal	*n* = 70, 100%	*n* = 70, 100%	*n* = 70, 100%	*n* = 67, 95.71%	χ^2^ = 9.097; *p* = 0.028
	Shortness grade I	*n* = 0, 0%	*n* = 0, 0%	*n* = 0, 0%	*n* = 3, 4.29%	
	Shortness grade II	*n* = 0, 0%	*n* = 0, 0%	*n* = 0, 0%	*n* = 0, 0.0 %	
Left PSLR	Normal	*n* = 70, 100%	*n* = 70, 100%	*n* = 70, 100%	*n* = 67, 95.71%	χ^2^ = 9.097; *p* = 0.028
	Shortness grade I	*n* = 0, 0%	*n* = 0, 0%	*n* = 0, 0%	*n* = 3, 4.29%	
	Shortness grade II	*n* = 0, 0%	*n* = 0, 0%	*n* = 0, 0%	*n* = 0, 0.0 %	
Pelvic tilt SR	Normal	*n* = 70, 100%	*n* = 70, 100%	*n* = 70, 100%	*n* = 62, 88.57%	χ^2^ = 24.70; *p* = 0.005
	Shortness grade I	*n* = 0, 0%	*n* = 0, 0%	*n* = 0, 0%	*n* = 6, 8.57%	
	Shortness grade II	*n* = 0, 0%	*n* = 0, 0%	*n* = 0, 0%	*n* = 2, 2.85%	
Distance SR	Normal	*n* = 70, 100%	*n* = 70, 100%	*n* = 70, 100%	*n* = 18, 25.71%	χ^2^ = 191.57; *p*<0.001
	Shortness grade I	*n* = 0, 0%	*n* = 0, 0%	*n* = 0, 0%	*n* = 29, 41.43%	
	Shortness grade II	*n* = 0, 0%	*n* = 0, 0%	*n* = 0, 0%	*n* = 23, 32.85%	
Pelvic tilt TT	Normal	*n* = 70, 100%	*n* = 70, 100%	*n* = 70, 100%	*n* = 46, 65.71%	χ^2^ = 63.34; *p*<0.001
	Shortness	*n* = 0, 0%	*n* = 0, 0%	*n* = 0, 0%	*n* = 24, 34.28%	
Distance TT	Normal	*n* = 70, 100%	*n* = 70, 100%	*n* = 70, 100%	*n* = 25, 35.71%	χ^2^ = 78.75; *p*<0.001
	Shortness grade I	*n* = 0, 0%	*n* = 0, 0%	*n* = 0, 0%	*n* = 22, 31.42%	
	Shortness grade II	*n* = 0, 0%	*n* = 0, 0%	*n* = 0, 0%	*n* = 23, 32.85%	

**Note:**

PSLR: passive straight leg raise test; SR: sit-and-reach test; TT: toe-touch test.

## Discussion

The objective of this study was to compare the extensibility of the hamstring musculature according to the dance style practiced in adolescent and young professional dance students. The main finding of this research was that the ballet dancers had higher degrees of hamstring extensibility than the Spanish and modern dancers. Another important outcome of this research was that the modern dancers had greater hamstring extensibility than the Spanish dancers.

A previous study also found this greater extensibility in adult professional ballet dancers (mean age: 30.70 ± 8.33 year-old) when compared to Folk dance (mean age: 32.94 ± 8.32 year-old) (Oreb et al., 2006). The differences in favor of the ballet dancers could be due to the fact that ballet dancers spend a long time stretching the hamstring musculature during their training as a consequence of the technique requirements (Oreb et al., 2006). A previous study has found that stretching volume influences hamstring extensibility (Santonja et al., 2007). Furthermore, dancers need an excellent concentric and eccentric control to properly perform a technique in movements which involve different execution speeds, also inducing an affect in hamstring extensibility (Grant, 2012). Along this line, the ballet technique has many steps in which the hamstring musculature is involved, such as *grand battement en avant and en arrière; battement développé en avant; souplé; penché, penchée en avant; and grand jeté en avant and grand jeté développé en avant*. These static or dynamic steps are characterized by the leg raise or by maximal trunk flexion with knee extension (Grant, 2012). Although some of these are also used in the other dance styles, their foundation lies in ballet, where they are most practiced (Forczek, Baena-Chicón & Vargas-Macías, 2017; Grant, 2012; Villanueva, 1995). Thus, ballet dancers need significant hamstring extensibility to execute these steps correctly (Grant, 2012).

There are other possible explanations. The reason why Spanish dancers have less extensibility may be due to the rhythmic stomping footwork with heel-tapping shoe. A previous study has found that a reduction of the forefoot supporting surface and the heel supporting surface can increase the activities of hip flexors and hamstring muscles and limit the pelvic movement during trunk flexion (Esenyel et al., 2003; Yoo, 2014). Another study has found that heel-tapping shoes reduce the ankle plantar flexor muscle involvement (Esenyel et al., 2003). Flamenco dancers have shown muscle imbalance between dorsiflexors and plantar flexors, with the first group being stronger than the second one (Pedersen et al., 1999). This muscular imbalance results in that during rhythmic stomping footwork, there are whole-body vibrations that are absorbed by hip joint and flexor and extensor joint muscles, so reductions in these muscles can be induced (Bejjani et al., 1988; Forczek, Baena-Chicón & Vargas-Macías, 2017). In fact, flamenco dancers have been found to have lower articular ranges in the iliopsoas muscle, the rectus femoris muscle and the soleus muscle for the same reason (González et al., 2011). Furthermore, as a consequence of use heel-tapping shoe, the base position for the Spanish dancers is with a little knee flexion (Echegoyen, Aoyama & Rodríguez, 2013), so it is possible that hamstring extensibility is a little less important for a proper technique in Spanish dance than in the other styles.

The results of these findings indicate that the individual monitoring of hamstring extensibility could be appropriate for Spanish dancers. It is possible that some of them need to increase their extensibility to improve the execution of the techniques found in some of the modalities included in Spanish dance, especially in “escuela bolera” and Spanish-stylized dance, which are styles that require a high hamstring extensibility for their technical execution, as the technical execution of the steps is very similar to ballet dance (Del Real, 2018). A previous research study has found that the demands of the techniques required by Spanish dance may cause uncompensated movements in less gifted dancers, which could result in overuse injuries. In fact, it is the second style with a high overuse ratio, only after ballet, with the first position in hamstring muscle injury (Sobrino, De la Cuadra & Guillén, 2015), which can be associated with muscle flexibility (Lui et al., 2012). Thus, only in some cases, an individual specific intervention would be necessary. This intervention would have to include a periodic and intermittent (Donti et al., 2018; Lima et al., 2019) training program, based on aerobics, strength (Koutedakis et al., 2007) and flexibility training, with the enough frequency needed to induce adaptations (Santonja et al., 2007), and a duration of more than 8 weeks of stretching to produce changes in the muscular and tendon structures and not only in neuromuscular dynamics (Freitas et al., 2018).

The current study found that all the dancer groups showed higher hamstring extensibility than the non-dancer group. Similar results have been found in previous studies which compare dancers, aged 8 to 16 years (mean: 13.3 years), who participated in different types of dancing classes and non-dancers of similar age (Steinberg et al., 2006), young flamenco dancers with sedentary individuals (mean age: 22.12 ± 4.21 year-old) (Gómez-Lozano et al., 2012), modern dancers (Chatfield et al., 1990) or young Latin style professional dancer with non-dancers (mean age: 23.44 ± 2.75 year-old) (Muyor, Zemková & Chren, 2017).

Previous researchers have analyzed the score needed in the hamstring extensibility test that indicates a hamstring extensibility that is enough to allow performing the movements of daily life without uncompensated movements which can increase the risk of injury (López-Miñarro et al., 2008; Sáinz de Baranda et al., 2005; Sobrino, De la Cuadra & Guillén, 2015). These classifications established three categories: normal, shortness grade I and shortness grade II, although there is no upper limit for the values of these tests in the classifications (López-Miñarro et al., 2008; Sáinz de Baranda et al., 2005). A relevant finding of this research is that all the dancers had extensibility within ranges considered healthy, while non-dancers showed between 4.29 and 74.29% of shortness cases depending on the test.

The significant hamstring extensibility shown by the dancers in this study could be associated with a low passive stiffness of the hamstring musculature and a high stretch tolerance (Marshall, Cashman & Cheema, 2011). This could be because all the dancers had a generic technical background of four years before beginning the specialization in a dance style, while the non-dancers did not perform any kind of exercise to avoid the natural tendency of shortening of the hamstrings (Vaquero-Cristóbal et al., 2015). This finding is important, as the shortening of the hamstring musculature is related to an increase in thoracic kyphosis in standing position and lumbar curvature flattening and an increase in posterior pelvic tilt in trunk flexion positions (López-Miñarro, Muyor & Alacid, 2014; McGill, 2002). Adopting trunk flexion positions with low hamstring extensibility systematically increases the probability of lesions in the spinal column and lower limb (Cejudo et al., 2019; Henderson, Barnes & Portas, 2010; McGill, 2002; Witvrouw et al., 2001). However, a high hamstring extensibility allows reaching maximal trunk flexion (López-Miñarro, Muyor & Alacid, 2014), which explains the high incidence of pain in the musculoskeletal system of the lower back and knee injuries in dancers (Lampe et al., 2019). It can thus be suggested that, although multi-component prevention programs are the most effective interventions for reducing sport-related injuries (Lauersen, Bertelsen & Andersen, 2014), core strength and stability training as a single factor has demonstrated to improve biomechanics and neuromuscular control, and stability of the lower limbs, reducing on its own the risk of injury in the lower back and lower limbs (De Blaiser et al., 2018; Dello Iacono, Padulo & Ayalon, 2016; Sasaki et al., 2019; Swain & Redding, 2014). In fact, core training program improves core muscles, balance and dance performance in dancers (Watson et al., 2017).

However, a note of caution is needed here, due to the heterogeneity of the dancers’ age included in the studies ranged from child to adult. Hamstring flexibility is influenced by age (Cejudo et al., 2019). Flexibility seems to decline with age as a consequence as joint capsule changes, for example, collagen increases in solubility, becomes more cross-linked; tendon or muscle stiffening (Adams, O’Shea & O’Shea, 1999). Early childhood and adolescence are critical periods for hamstring extensibility due to the differential femur growth in relation to muscle length, which can result in a decrease in hamstring extensibility (De Ste Croix & Korff, 2013). However, this tendency can be different in disciplines where hamstring extensibility is essential for the performance of the techniques. In fact, in aesthetic group gymnastics, it has found that the oldest adolescents showed higher hamstring extensibility than the youngest, which can be a result of the adaptation of technical necessities (Conesa-Ros, Martínez-Gallego & Santonja, 2016). Along this line, the findings of the current study have shown that age was a significant covariable for pelvic tilt in SR. Furthermore, positive correlations were found between age and pelvic tilt in SR in Spanish and modern dancers, and between age and the hip flexion in ASLR test with left leg in modern dancers. However, age was not a covariable for the rest of the tests and no significant correlations were found in the other tests and in the ballet group. Thus, further work is required for establishing the evolution of hamstring extensibility according to age in dancers.

A much debated question is if BMI can influence hamstring extensibility. Previous studies in schoolchildren aged from 12 to 18 years old have suggested that there are no differences in hamstring extensibility according to BMI (Deforche et al., 2003; Tokmakidis, Kasambalis & Christodoulos, 2006). However, other research studies have found that there are differences in hamstring extensibility according with BMI in girls aged from 13 to 18.5 years. Underweight girls showed lower weight values, although this group had higher flexibility values, without differences between the other categories (Artero et al., 2010). Interestingly, BMI was not a covariable in any hamstring test in the present study. However, a negative correlation was found between BMI and ASLR tests and PSLR test with left leg in ballet group; while a positive correlation was found between BMI and pelvic tilt in SR in Spanish and non-dancer groups. According with Artero et al. (2010), a relationship between BMI and hamstring extensibility cannot be linear. Therefore, the differences in the direction of the correlation among ballet, Spanish and non-dancers can be due to ballet showing the lowest BMI values. However, BMI does not allow for distinguishing between fat and muscle masses (Herrero & Cabañas, 2009) and fat mass has been shown to be the main factor that determines the relationship between BMI and hamstring extensibility (Artero et al., 2010). Therefore, further work is required for establishing the relationship between body composition and hamstring extensibility in dancers.

The practice of dance can be influenced by hamstring extensibility, and a stronger focus on the evolution of hamstring extensibility and spine disposition related to progressive specialization in a dance style according to academic year, age, BMI and body composition is necessary. It would be convenient to carry out future research analyzing the effect of an injury prevention program for dancers on injuries and the influence in hamstring extensibility and spine curvatures. Furthermore, future studies on the training to improve hamstring extensibility of Spanish dancers with less extensibility are therefore recommended.

## Conclusions

The systematic practice of dance, regardless of the style, leads to high ranges of hamstring extensibility. The lack of specific training of the hamstring extensibility could lead to different grades of shortness, as it has been observed in the control group. Ballet dancers have greater hamstring extensibility than the dancers of the other styles, followed by those of modern dance and lastly, Spanish dance. The differences were found in both active and passive tests and could be consequence of the techniques required in this dance style.

## Supplemental Information

10.7717/peerj.9237/supp-1 Supplemental Information 1 Database.

## Additional Information and Declarations

### Data Availability

The following information was supplied regarding data availability: Raw data is available as a Supplemental File.
